# Biological Trajectory of Virophage Research and the Emergence of Marine Virophages: A Scoping Review

**DOI:** 10.3390/v18050560

**Published:** 2026-05-14

**Authors:** Min-Jeong Kim, Yu Jin Kim, Hyun Ju Ha, Joon Sang Park, Ika Agus Rini, Sukchan Lee, Taek-Kyun Lee

**Affiliations:** 1 Ecological Risk Research Department, Korea Institute of Ocean Science & Technology, Geoje 53201, Republic of Korea; min-jeong214@kiost.ac.kr (M.-J.K.); rladbwls06069@kiost.ac.kr (Y.J.K.); 2 Department of Ocean Science, University of Science and Technology, Daejeon 34113, Republic of Korea; calltolove@kiost.ac.kr (H.J.H.); jspark1101@kiost.ac.kr (J.S.P.); 3 Library of Marine Samples, Korea Institute of Ocean Science & Technology, Geoje 53201, Republic of Korea; 4 Department of Integrative Biotechnology, Sungkyunkwan University, Suwon 16419, Republic of Korea; ikaagusrini@g.skku.edu (I.A.R.); cell4u@skku.edu (S.L.)

**Keywords:** virophage, marine virophages, giant viruses, metagenomics, research trends

## Abstract

Virophages are satellite viruses that depend on the replication machinery of giant double-stranded DNA viruses and influence the structure and dynamics of viral communities through multilayered interactions among giant viruses, their hosts, and virophages. Since the discovery of the Sputnik virophage in 2008, virophages have been increasingly recognized for their roles in regulating giant virus replication, contributing to host defense mechanisms, and shaping the evolution of mobile genetic elements. However, quantitative syntheses examining how virophage research has developed over time, particularly in marine environments, remain limited. Here, we conducted a bibliometric analysis of virophage research published between 2008 and 2025 using the Web of Science Core Collection. By comparing an overall virophage research corpus with a marine virophage sub-corpus, we assessed publication and citation trends, collaboration structures, and keyword-based intellectual and thematic evolution. Our results show that virophage research has gradually transitioned from an early phase dominated by landmark discoveries and experimental model systems to a data-intensive stage driven by genome- and metagenome-based analyses and computational approaches. Although marine virophage studies represent a relatively small proportion of the total literature, they exhibit sustained citation impact and form a distinct research axis within the field. In particular, marine-focused studies emphasize metagenomic discovery, genome sequence alignment, and the analysis of mobile genetic elements such as polinton-like viruses, highlighting the role of marine environments in accelerating the intellectual transition of virophage research. Collectively, these findings demonstrate that virophage research has moved beyond a “discovery and definition” phase toward data-driven integrative interpretation, with marine virophage research emerging as a key domain for understanding the structure and evolutionary dynamics of marine viral ecosystems.

## 1. Introduction

Virophages are satellite viruses that depend on the replication machinery of giant double-stranded DNA (dsDNA) viruses and represent unique biological entities capable of reshaping viral ecosystems through complex virus–virus–host interactions [1,2,3]. Since the discovery of the Sputnik virophage in 2008 from Acanthamoeba infected by Mimivirus-like giant viruses, virophages have emerged as key biological factors that extend beyond the classical concept of satellite viruses. Accumulating evidence indicates that virophages can modulate giant virus replication, contribute to host defense strategies, and participate in the evolution of mobile genetic elements, thereby influencing broader patterns of viral and host evolution [1,2].

Early investigations of virophages were largely grounded in cultivation-based experimental systems, focusing on giant virus–amoeba host models to elucidate their molecular features, replication strategies, and inhibitory effects on giant virus proliferation [4]. These studies played a central role in defining the biological identity of virophages and establishing their mechanistic relevance within tripartite virus–virus–host systems. However, as metagenomic technologies rapidly advanced, it became evident that virophages and evolutionarily related genetic elements—particularly polinton-like viruses (PLVs)—are not restricted to laboratory model systems. Instead, they are widespread across natural environments and form part of a broader evolutionary continuum linking eukaryotic viruses, transposable elements, and host genomes [5,6,7,8].

Marine ecosystems provide a particularly important context for expanding virophage research. Oceans harbor vast populations of giant viruses and diverse eukaryotic microbial hosts, creating ecological conditions that favor complex viral interactions and large-scale genetic exchange [9,10,11]. Global marine metagenomic surveys have repeatedly reported virophages or virophage-like sequences across a wide range of marine environments, including open oceans, coastal waters, and polar regions [5,6]. These findings suggest that virophages may influence the structure and dynamics of marine viral communities, contributing to horizontal gene transfer and long-term evolutionary processes in marine ecosystems [12,13]. Despite recent advances, biological research on marine virophages remains limited because only a small number have been isolated, limiting detailed experimental characterization and broader biological interpretation.

Although knowledge of virophage biology and ecology has expanded substantially, most existing syntheses have focused on taxonomic classification, molecular mechanisms, or evolutionary hypotheses through narrative reviews [3,7]. Consequently, a field-wide perspective that integrates how experimental systems, environmental discovery, and genome-based analyses have collectively shaped the biological understanding of virophages over time is still lacking. In particular, the extent to which marine studies have driven conceptual transitions—from model organism–based investigations toward genome-centered and ecosystem-scale interpretations—has not been clearly articulated.

In this study, we conduct a scientometric analysis of virophage research since its first report in 2008. Using publications indexed in the Web of Science Core Collection, we examine patterns in research activity, collaboration, and thematic focus to understand how the virophage research field has developed over time. By mapping publication patterns, citation structures, and keyword networks, this study aims to provide a structural overview of the intellectual organization and thematic evolution of virophage research. By explicitly distinguishing between the broader virophage literature and marine-focused studies, we highlight marine environments as key arenas where genome-based discovery, comparative analysis, and evolutionary interpretation have converged. Rather than presenting a conventional narrative review of virophage biology, this study focuses on quantitatively mapping the intellectual structure and developmental trajectory of the research field.

## 2. Materials and Methods

### 2.1. Data Collection

The bibliometric analysis in this study used the Web of Science (WoS) Core Collection as the primary data source. WoS provides systematically structured bibliographic information, including authors, affiliations, countries, journals, citation data, Author Keywords, and Keywords Plus, and is widely used for science mapping, collaboration network analysis, and the analysis of thematic evolution in scientific research [14,15]. Literature retrieval was performed using the Topic (TS) field in WoS, which encompasses the title, abstract, Author Keywords, and Keywords Plus.

To comprehensively capture studies on virophages and related concepts, a two-stage search strategy was used. First, virophage-related keywords were used to retrieve the global corpus of virophage research from the Web of Science Core Collection. Subsequently, marine-related keywords were applied to identify studies on marine environments, which were compiled into a marine virophage sub-corpus for further analysis. The search query used to build the full dataset was as follows: TS = (virophage OR “viro-phage” OR “virophage-like” OR “Sputnik virus” OR Mavirus OR “transpovirion” OR “Polinton-like virus” OR “Polinton virus*”).

The marine virophage research corpus was derived by extending the above virophage search query with additional keywords reflecting marine environments, water mass characteristics, and metagenomic approaches. This strategy enabled the systematic separation of marine-based virophage studies from the overall literature. The search query used to construct the marine sub-corpus was: TS = ((virophage OR “viro-phage” OR “virophage-like” OR “Sputnik virus” OR Mavirus OR “transpovirion” OR “Polinton-like virus” OR “Polinton virus*”) AND (marine OR ocean* OR seawater OR pelagic OR estuar* OR plankton OR coastal OR shelf OR virome* OR metagenome*)).

In Web of Science search syntax, quotation marks were used for exact phrases or fixed multi-word expressions, while single-word terms were searched without quotation marks to broaden the search. The asterisk (*) served as a wildcard operator to capture word variations sharing the same root.

The literature search was conducted on 6 January 2026. Retrieved records were refined through a stepwise screening process based on document type, language, duplication status, and topical relevance. The final dataset comprised publications published between 2008 and 2025 that were retrievable in the Web of Science Core Collection at the time of the search. The confirmed overall virophage corpus and the marine virophage sub-corpus were used for all subsequent analyses. No review protocol was registered for this scoping review, and a registration number is therefore not applicable.

### 2.2. Screening and Exclusion Criteria

A stepwise eligibility screening procedure was applied to all retrieved records. First, non-research document types, including Proceedings Papers, Corrections, Letters, Meeting Abstracts, and News Items, were excluded. Second, the title, abstract, Author Keywords, and Keywords Plus were examined to remove records that were not relevant to the predefined virophage-related search terms. Records were retained when one or more of the predefined search terms were identified in these searchable fields and were considered relevant to the scope of this bibliometric analysis. Articles were excluded when the retrieved terms were clearly unrelated to virophage research in context or when the record did not fit the study’s thematic scope, as determined through manual screening. Third, duplicate records sharing identical DOIs or bibliographic information (title, authors, publication year, and journal) were removed. For the marine virophage sub-corpus, studies lacking a clear and explicit association with marine environments—such as seawater, coastal zones, open oceans, plankton, or marine metagenomes—were excluded from further analysis (Table S1). The literature retrieval and screening workflow was designed with reference to PRISMA flow diagram principles, and the search and refinement process was reported transparently to ensure analytical reproducibility and methodological reliability [16]. A critical appraisal of the included sources was not performed, as the aim of this scoping review was to map the scope, characteristics, and thematic development of the literature rather than to assess study quality for evidence synthesis.

### 2.3. Data Extraction and Preprocessing

Bibliographic records were exported from WoS in the “Full Record and Cited References” format and subsequently imported into the R statistical environment. The records were converted into analysis-ready data frames using the bibliometrix package [17]. The primary metadata fields used in the analyses included publication year, journal, authors, affiliations, countries, citation indicators (total citations, TC), Author Keywords (DE), Keywords Plus (ID), and cited references (CR). Data charting was conducted using a predefined extraction framework developed for this review. The extracted bibliographic information was checked for consistency and completeness during preprocessing, and any discrepancies were resolved by reviewing the original Web of Science records.

To ensure accuracy and consistency in keyword-based analyses, a stepwise keyword standardization procedure was applied to both Author Keywords and Keywords Plus. First, variations in capitalization and singular–plural forms were unified to minimize redundant counting of identical concepts. Second, differences arising from the use of hyphens or spacing were normalized into a single standardized term (e.g., viro-phage to virophage). Third, semantically equivalent terms were consolidated under unified categories; for example, NCLDV and nucleocytoplasmic large DNA viruses were treated as a single concept.

For specific visualization analyses, such as word clouds, highly dominant core terms with disproportionate semantic weight (e.g., virophage and its variants) were selectively excluded to facilitate clearer comparisons of relative thematic differences. Conversely, these terms were retained in network-based analyses where keyword relationships and centrality interpretations were required. This combination of keyword standardization and selective exclusion follows widely adopted best practices in bibliometric keyword analysis [15,18].

### 2.4. Descriptive Statistics and Citation Metrics

Descriptive statistical analyses were conducted to calculate the total number of publications, temporal coverage, total citations (TC), mean citations per article, and the average number of publications per year for each corpus. Annual publication trends and citation dynamics were aggregated by publication year and visualized in time-series formats to enable direct comparison between the overall virophage research corpus and the marine virophage sub-corpus.

Journal distribution analysis was performed based on the number of publications per journal, and both absolute publication counts and relative contributions (%) to the overall corpus were calculated. Citation indicators were derived from raw cumulative citation counts retrieved from the Web of Science Core Collection and were used as descriptive indicators of cumulative scholarly visibility within the retrieved corpus. Citation normalization by publication year was not applied because the aim of this study was to describe long-term research growth and historical citation patterns, rather than year-adjusted article-level impact.

The relative contribution of marine virophage research was evaluated by calculating the annual proportion of marine-related publications relative to the total number of virophage publications using the following formula: (number of marine virophage articles /total number of virophage articles) × 100. The selection of indicators and analytical procedures followed standard practices commonly adopted in bibliometric studies [14,17].

### 2.5. Social Structure Analysis

National research contributions were quantified based on the affiliation countries of authors listed in each publication. For each country, both the absolute number of publications and their proportional shares were calculated. International collaboration networks were constructed by defining a collaborative link when authors affiliated with different countries co-authored the same publication. Author collaboration networks were constructed from co-authorship relationships, with authors represented as nodes and co-authorship ties as edges.

Network calculations and metric derivations were conducted using the bibliometrix package in R, while VOSviewer (version 1.6.20) was used as a complementary tool to enhance network visualization and the presentation of clustering results [19]. Network clustering was performed using an association strength–based algorithm.

Research leadership was assessed based on the affiliations of first and corresponding authors, using author order and corresponding author information from Web of Science records [20,21,22]. In addition, the top 20 most cited articles were identified based on total citation counts (TC) from Web of Science. For both the overall virophage corpus and the marine virophage sub-corpus, the thematic characteristics, leading contributing countries, and journal distributions of these highly cited publications were compared.

### 2.6. Intellectual Structure and Thematic Evolution

Intellectual structure analysis was primarily conducted using co-word analysis. Keyword co-occurrence networks were constructed from Author Keywords and Keywords Plus, with keywords represented as nodes and co-occurrence within the same publication as links [18]. Major research themes were interpreted by jointly considering keyword frequency and network centrality metrics.

Thematic maps were generated using a centrality–density framework. In this framework, centrality reflects the degree of interaction with other themes and indicates thematic relevance, whereas density represents internal cohesion and reflects thematic development or maturity. Based on these measures, themes were classified into motor, basic, niche, and emerging or declining themes [18,23]. The same analytical procedures were independently applied to both the overall virophage corpus and the marine virophage sub-corpus to enable direct comparison of their thematic structures.

Thematic evolution analysis was performed by dividing the entire study period into multiple time slices and visualizing the continuity and transitions among thematic clusters across successive periods using Sankey diagrams. The thickness of the connecting flows represents the degree of keyword overlap between adjacent periods, allowing the emergence, persistence, diversification, and transformation of research themes to be interpreted along a temporal axis [17,18].

### 2.7. Topic Trends and Country–Keyword Coupling

Topic trend analysis was conducted by visualizing the temporal ranges of occurrence, central appearance years (mean or median), and frequencies of major keywords. This approach enabled the differentiation between topics that were prominent during early stages of the field and those that have emerged more recently [17].

Country–keyword coupling analysis was performed based on co-occurrence relationships between authors’ affiliated countries and keywords, allowing assessment of thematic specialization and research focus across countries [15,17,23]. The results were visualized using chord diagrams. To enhance interpretability, the analysis focused on countries with high publication contributions and keywords with high occurrence frequencies.

### 2.8. Software and Reproducibility

All bibliometric indicators and analyses were conducted in the R (version 4.5.1) statistical environment. Primary analyses and visualizations were performed using the bibliometrix package and its biblioshiny interface [17]. VOSviewer was used as a complementary tool to enhance the visualization and clustering of collaboration networks and keyword co-occurrence networks [19].

Both the overall virophage research corpus and the marine virophage sub-corpus were processed using identical data extraction, preprocessing, analytical, and visualization pipelines, ensuring direct comparability and internal consistency across datasets. The analytical workflow was designed to follow the standard procedures implemented in bibliometrix, thereby ensuring reproducibility and methodological reliability of the results.

## 3. Results

### 3.1. Data Collection and Descriptive Characteristics

**Figure 1:**
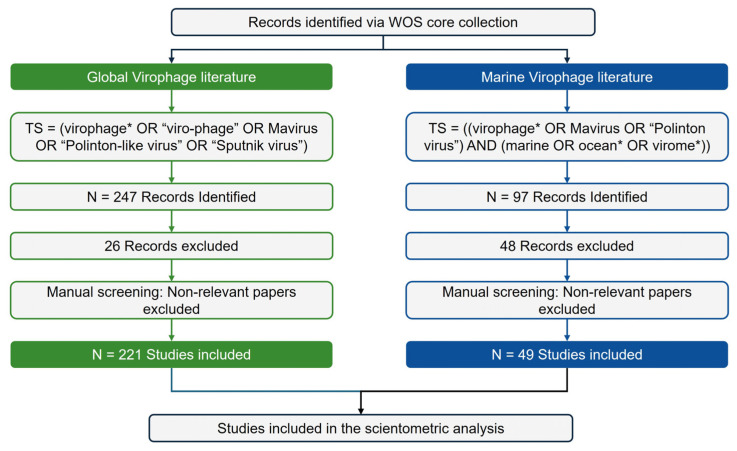
PRISMA-based systematic workflow for corpus construction and data refinement. A multi-stage flow diagram delineating the identification, screening, and inclusion process for the Global Virophage Corpus (GVC) and the Marine Virophage Sub-corpus (MVC).

Application of predefined search queries to the Web of Science Core Collection (WoSCC) initially returned 247 publications on virophage research. After stepwise refinement based on document type (e.g., proceedings papers, corrections), topical relevance, and duplication status, a final set of 221 publications was confirmed as the overall virophage research corpus (Figure 1; Table S1). The earliest record retrieved from the dataset dates back to 2008, reflecting early studies on genetic elements evolutionarily related to virophages. For the marine virophage subdomain, the application of additional marine-related search terms yielded 97 publications in the initial retrieval. After excluding studies lacking explicit analysis of marine environments or marine-derived datasets, as well as those with low topical relevance, 49 publications were ultimately selected for analysis.

The overall virophage corpus (*n* = 221) accumulated 10,434 citations over the analysis period from 2008 to 2025, with a mean of 47.21 citations per article (Table 1). The average annual publication output was 12.3 articles. The marine virophage corpus (*n* = 49) accounted for 22.2% of the total literature and accumulated 1642 citations, with a mean of 33.5 citations per article (Table 1). The average annual publication output for the marine subdomain was 2.7 articles.

### 3.2. Research Growth Phases and Citation Dynamics

**Figure 2:**
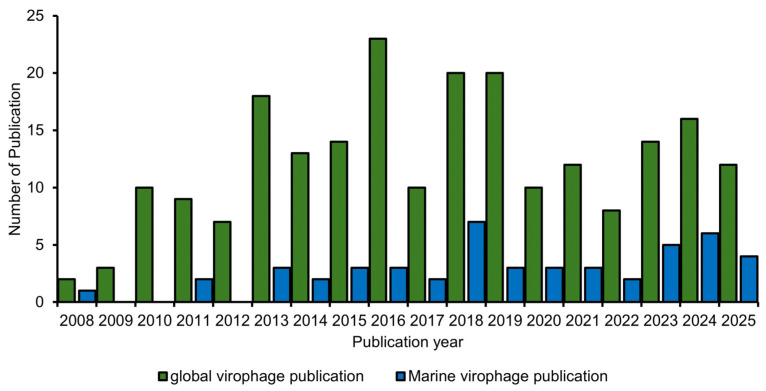
Annual publication trends and growth dynamics of virophage research (2008–2025). Temporal distribution of annual research output for the Global Virophage Corpus (GVC; green line) and the Marine Virophage Sub-corpus (MVC; blue area).

Analysis of annual publication trends from 2008 to 2025 revealed distinct phases in the development of virophage research (Figure 2). In 2008, publication output increased gradually, and from approximately 2014 onward, annual output stabilized at around 20 publications per year (Figure 2).

The annual publication pattern of marine virophage research generally followed the overall trend observed across the corpus; however, absolute publication counts remained in the single-digit range in most years. Over several years after 2018, the number of publications on marine virophages increased. The proportion of marine virophage studies relative to the total virophage literature varied across years as overall publication output increased (Figure S1), with marine-related publications accounting for approximately 30–40% of the total output in some years.

Analysis of annual citation counts showed that citations for the overall virophage corpus increased markedly from the late 2000s onward, with more than 1639 citations recorded in some years during the mid-2011s (Figure S2). Thereafter, annual citation counts alternated between increases and decreases. Annual citation counts for marine virophage publications were consistently lower than those of the overall corpus; however, their temporal fluctuation patterns were similar, with relatively higher citation counts observed in some years around 2008 (Figure S2).

**Figure 3:**
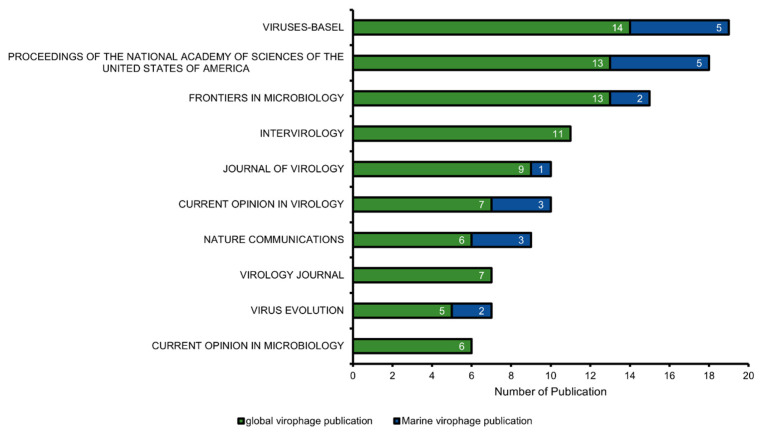
Journal-wise distribution and comparative analysis of virophage research publications. A comparative visualization of publication counts across the top 10 journals for the Global Virophage Corpus (GVC; green) and the Marine Virophage Sub-corpus (MVC; blue).

Journal distribution analysis indicated that virophage research was disseminated across a wide range of virology and microbiology journals, although publication output was concentrated in a limited number of outlets (Figure 3). The leading journals for overall virophage research were Viruses (Basel), Proceedings of the National Academy of Sciences of the United States of America, Frontiers in Microbiology, and Intervirology (Figure 3). Marine virophage studies showed a similar distribution pattern, with relatively higher numbers of publications appearing in Viruses (Basel), Frontiers in Microbiology, and PNAS (Figure 3).

### 3.3. Social Structure and Research Leadership

Analysis of country-level publication contributions showed that the overall virophage research corpus was characterized by a concentrated distribution across a limited number of countries (Table 2). France contributed the largest number of publications with 103 articles (46.2%), followed by the United States with 66 articles (29.6%) and Germany with 36 articles (16.1%). Brazil and China each contributed 17 articles (7.6%), while the United Kingdom (15 articles, 6.7%), Canada (13 articles, 5.8%), Japan (11 articles, 4.9%), the Netherlands (11 articles, 4.9%), and Poland (9 articles, 4.0%) completed the top ten contributing countries.

Within the marine virophage corpus (*n* = 49), the United States ranked first with 16 publications (32.7%), followed by Germany with 13 publications (26.5%) and France with 11 publications (22.4%). China, the United Kingdom, and the Netherlands each contributed 7 publications (14.3%). Japan (5 publications, 10.2%), Canada (3 publications, 6.1%), Poland (2 publications, 4.1%), and Brazil (1 publication, 2.0%) accounted for smaller shares of the marine subdomain. In addition to the top ten countries, smaller contributions were identified from several other countries.

**Figure 4:**
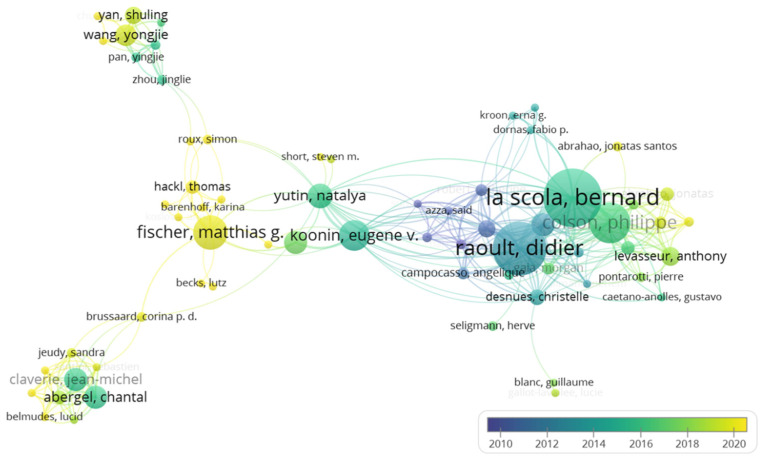
Author co-authorship network and temporal evolution in virophage research. A network visualization illustrating the collaborative landscape and the chronological development of the virophage research community.

The author’s co-authorship network analysis indicated that overall virophage research exhibited a hub-and-spoke structure centered on a limited number of core researchers (Figure 4). The central region of the network was occupied by Bernard La Scola, Didier Raoult, Philippe Colson, and their collaborators, who were highly connected. Eugene V. Koonin and Natalya Yutin were positioned as connectors linking the central hub to peripheral research groups (Figure 4). Additional collaboration clusters were observed around Matthias G. Fischer and Thomas Hackl, as well as around Jean-Michel Claverie and Chantal Abergel. Overlay visualization by publication year indicated that research activity within the central hub was primarily concentrated in the early to mid-2010s, whereas more recent publication activity was observed in some peripheral clusters.

**Figure 5:**
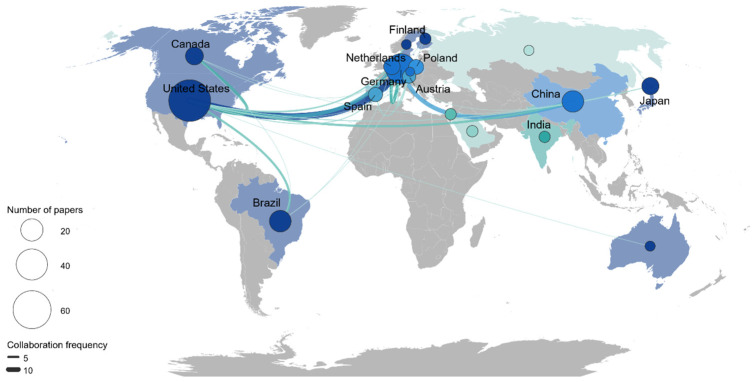
Global landscape of international collaboration in virophage research. A spatial visualization of international co-authorship patterns, illustrating the degree of scientific integration across borders.

International collaboration networks at the country level were organized primarily around North America and Europe (Figure 5). The United States and France were the largest nodes, reflecting both high publication output and extensive international collaboration. Numerous connections were observed with European countries, including Germany, the United Kingdom, the Netherlands, and Italy. In Asia, China and Japan appeared as relatively large nodes, while Canada and Brazil were represented as independent nodes in North and South America, respectively, with confirmed collaborative links to the United States and European countries (Figure 5).

Analysis of the distributions of corresponding and first authors by country showed that corresponding authors were predominantly concentrated in North America and Europe, with the United States hosting the largest number (Figure S3). Germany, France, the United Kingdom, the Netherlands, and Canada also exhibited relatively high numbers of corresponding authors. The spatial distribution of first authors closely resembled that of corresponding authors; however, in some countries, the number of first authors exceeded that of corresponding authors. In Asia, China and Japan accounted for relatively large numbers of first authors, while Brazil emerged as a major contributor in South America (Figure S3).

Based on total citation counts (TC), the top 20 most cited articles in the overall virophage corpus ranged from 108 to 780 citations (Table S2). The most highly cited article was “VirSorter2: a multi-classifier, expert-guided approach to detect diverse DNA and RNA viruses,” published in 2021, which accumulated 780 citations. These highly cited articles were published across a diverse set of journals, including Nature, Microbiome, Viruses, and Nature Reviews Microbiology.

In the marine virophage corpus, the top 20 most cited articles accumulated between 14 and 425 citations (Table S3). The most highly cited article was “The virophage as a unique parasite of the giant *mimivirus*,” published in 2008, with 425 citations. These articles appeared in journals such as Nature, Proceedings of the National Academy of Sciences of the United States of America, and mBio.

### 3.4. Intellectual Structure and Thematic Evolution

**Figure 6:**
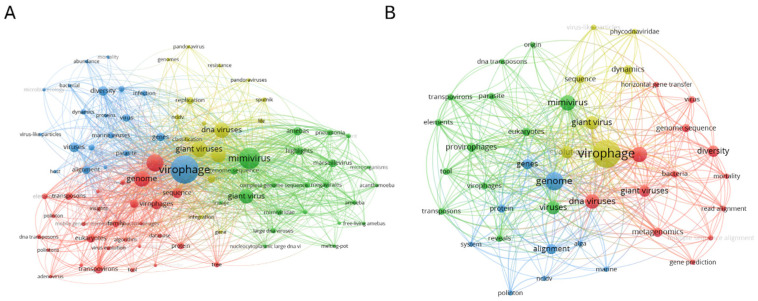
Keyword co-occurrence networks and intellectual structure of virophage research. Comparative visualization of keyword co-occurrence for (**A**) the Global Virophage Corpus (GVC) and (**B**) the Marine Virophage Sub-corpus (MVC).

Keyword co-occurrence network analysis revealed that the overall virophage corpus was organized into multiple thematic clusters (Figure 6A). At the center of the network, virophages showed high co-occurrence with giant viruses, *mimiviruses*, the *mimivirus* genome, and DNA viruses (Figure 6A). The green cluster comprises *mimiviruses, giant viruses, amoebae, Acanthamoeba, and Marseilleviruses*. The red cluster includes virophage, *transpovirus,* eukaryotic, and DNA transposon genomes. The yellow cluster consists of replication, genes, genomes, and resistance, whereas the blue cluster includes viruses, diversity, marine viruses, bacteria, and dynamics.

A multi-cluster structure was also observed in the marine virophage corpus (Figure 6B). In this network, virophage was strongly connected to the giant virus, *mimivirus*, genome, and DNA viruses, functioning as an inter-cluster linking node. The green cluster included *mimivirus*, giant virus, eukaryotes, and genes. The red cluster consisted of diversity, metagenomics, bacteria, and horizontal gene transfer. The blue cluster comprised alignment, protein, polinton, and virus, whereas the yellow cluster was centered on virophage, genome, and DNA viruses.

Keyword frequency analysis showed that virophage appeared in 51 publications (34.0%) across the corpus as a whole (Table S4). Giant viruses and *Mimivirus* were each identified in 32 publications (21.3%), followed by giant viruses (19 publications, 12.7%) and virophages (18 publications, 12.0%). Other terms, such as Megavirales, *Marseillevirus*, NCLDV, and *Mimiviridae*, also appeared at relatively high frequencies. In the marine corpus, virophage appeared in 8 publications (22.9%), while metagenomics and diversity each appeared in 4 publications (11.4%). Giant virus, virus/viruses, NCLDV, polinton, and horizontal gene transfer were identified in 3–4 publications.

Word cloud analysis based on Author Keywords further illustrated differences between the two corpora (Figure S4). In the overall corpus, virophage, giant virus, *mimivirus*, NCLDV, and virus evolution were visually prominent (Figure S4A). In contrast, the marine corpus was characterized by the prominence of virophages, protists, plankton, metagenomics, giant viruses, and NCLDVs (Figure S4B).

**Figure 7:**
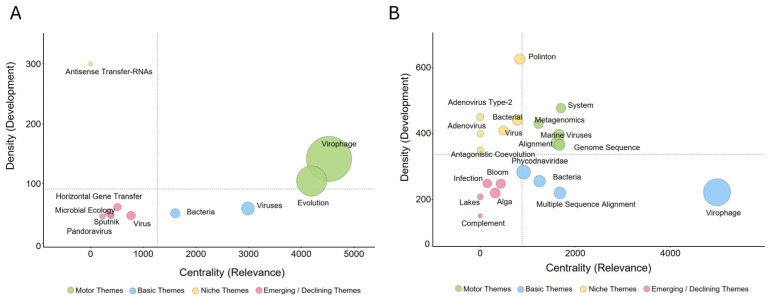
Strategic diagrams and thematic maturity of virophage research. Thematic maps based on centrality and density for (**A**) the Global Virophage Corpus (GVC) and (**B**) the Marine Virophage Sub-corpus (MVC).

Thematic map analysis positioned virophage and Evolution in the overall corpus as a basic theme with high centrality and moderate density (Figure 7A). Bacteria and viruses also exhibited relatively high centrality. In the marine corpus, virophage similarly showed the highest centrality and was classified as a basic theme (Figure 7B). Genome sequence, alignment, marine viruses, and bacteria displayed moderate to high centrality and density, whereas polinton exhibited high density but low centrality (Figure 7B).

**Figure 8:**
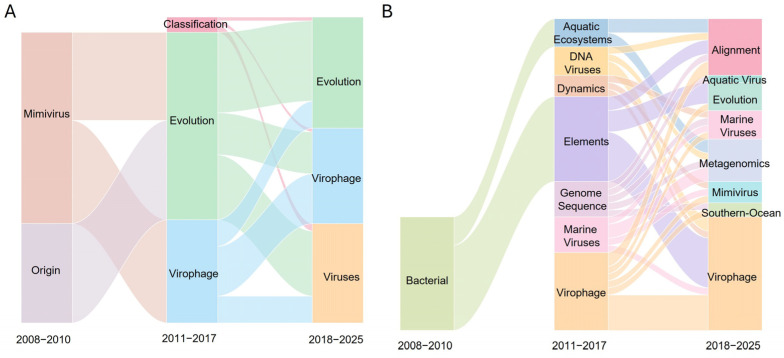
Thematic evolution and transition pathways in virophage research. Sankey diagrams illustrating the longitudinal evolution of research themes for (**A**) the Global Virophage Corpus (GVC) and (**B**) the Marine Virophage Sub-corpus (MVC) across three strategic periods: 2001–2010, 2011–2017, and 2018–2025.

Thematic evolution analysis indicated that, in the overall corpus, *Mimivirus* and Origin were dominant themes during 2008–2010, followed by the emergence of Classification, Evolution, and Virophage during 2011–2017 (Figure 8A). During 2018–2025, Evolution, Virophage, and Viruses were identified as major themes (Figure 8A). In the marine corpus, bacteria emerged as a dominant theme during 2008–2010. During 2011–2017, Aquatic Ecosystems, DNA viruses, Dynamics, Elements, Genome sequence, Marine viruses, and Virophage were identified, while the 2018–2025 period was characterized by Alignment, Aquatic virus Evolution, Marine Viruses, Metagenomics, *Mimivirus*, Southern-Ocean, and Virophage (Figure 8B).

Topic trend analysis showed that, in the overall corpus, origin, amoebas, and *Mimivirus* were prominent in the early period, followed by eukaryotes, evolution, *Marseillevirus,* and giant virus during the intermediate period. In the most recent period, virophage, virus, provirophage, and alignment were identified (Figure S5A). In the marine corpus, dynamics, genome sequences, marine viruses, *mimiviruses*, giant viruses, metagenomics, bacteria, and elements appeared relatively early. More recently, virophage, DNA viruses, provirophage, eukaryotes, evolution, viruses, and alignment were identified (Figure S5B).

Country–keyword coupling analysis showed that, in the overall corpus, virophage, giant virus, *mimivirus*, polinton, and metagenomics co-occurred with a wide range of countries (Figure S6A). In the marine corpus, virophage, marine viruses, metagenomics, viruses, and antiviral defense showed co-occurrence across multiple countries (Figure S6B).

## 4. Discussion

### 4.1. Overview of the Study and Key Findings

Using a Web of Science Core Collection–based bibliometric approach, this study quantitatively examined how virophage research has developed and expanded between 2008 and 2025, focusing on its growth trajectory, social collaboration structure, and intellectual evolution (Figure 1; Table 1 and Table S1). By separately constructing and comparing an overall virophage corpus (*n* = 221) and a marine virophage sub-corpus (*n* = 49), we structurally demonstrated how an early research axis centered on experimental model systems following the discovery of the Sputnik virophage has coexisted with, and gradually transitioned toward, a data- and computation-driven research axis associated with the expansion of environmental metagenomics [1,17,19].

From a descriptive perspective, the overall corpus accumulated a total of 10,434 citations (mean = 47.21 citations per article), whereas the marine sub-corpus accumulated 1642 citations (mean = 33.5 citations per article) (Table 1). Despite its relatively small publication volume, the marine subdomain maintained a mean citation impact comparable to that of the overall corpus, indicating that it has achieved distinct scholarly visibility and influence within a limited-scale literature (Table 1). These results support the view that marine virophage research has functioned not merely as an applied or peripheral subtopic, but as a key axis facilitating the broader transition of virophage research through metagenomics-based discovery and evolutionary interpretation [6,7].

### 4.2. Growth and Transition of Virophage Research: Sustained Accumulation Beyond Initial Discovery

Annual publication trends (Figure 2) show that virophage research exhibited followed by a pronounced growth phase after 2008 (Figure 2). Publication output increased steadily after 2010, exceeding 20 articles per year in several years, and annual citation activity expanded markedly, with some years after the mid-2011s recording more than 1000 citations (Figure S2). These patterns indicate that virophage research did not emerge as a short-lived topic driven by a single discovery event, but rather matured into a stable research field through sustained accumulation of follow-up studies and methodological diversification.

This growth transition can be interpreted as the combined outcome of at least three factors. First, the discovery of the Sputnik virophage introduced a novel conceptual category—viruses that parasitize the replication machinery of giant viruses—thereby establishing a conceptual threshold for subsequent research [1]. Second, as tripartite interactions among giant viruses, hosts, and virophages became recognized as an independent biological question, virophages began to be viewed as objects of evolutionary and ecological significance rather than as simple satellite viruses [2,3]. Third, rapid advances in genomic and metagenomic technologies provided the technical foundation for detecting and analyzing virophages and related genetic elements across diverse environmental samples, overcoming the limitations of cultivation-based approaches [4,7]. Together, conceptual innovation, the emergence of independent research questions, and technological expansion appear to have interacted synergistically to sustain the field’s growth.

### 4.3. Marine Virophage Research as an Observational Window Accelerating Intellectual Transition

Marine virophage research followed a temporal trajectory broadly similar to that of the overall field, although its annual publication output remained comparatively low (Figure 2). Nevertheless, during specific periods, it accounted for 20–30% of total virophage publications, and in some years reached approximately 30–40%, indicating that marine environment–based studies contributed substantially to the quantitative expansion of the field during key phases (Figure S1). After 2018, a relatively stable level of contribution was observed, suggesting that the marine subdomain has become established as an independent research axis (Figure S1).

The expansion of marine virophage research has been driven primarily by approaches that detect virophage or polinton-like virus (PLV) signals within large-scale environmental genomic datasets and interpret their evolutionary significance through annotation, classification, and phylogenetic inference [3,6]. In contrast to early cultivation- and isolation-based studies, this shift highlights the role of marine environments as observational windows that facilitate data-intensive, computation-centered transitions in virophage research. More recently, experimental studies reporting the isolation and infection cycles of PLV- or virophage-like agents in marine algal systems have begun to emerge, indicating early convergence between metagenomics-based inference and experimental validation [24]. Taken together, marine virophage research can be understood as a methodological and conceptual driver that advances virophage research through a sequence of environment-based discovery, computational classification, and evolutionary interpretation, and selective experimental verification.

### 4.4. Social Structure and Research Leadership: Advantages and Constraints of a Hub-Centered System

Country- and author-level analyses (Table 2; Figure 4 and Figure 5 and Figure S3) show that virophage research has developed a hub-centered social structure led by a small number of countries and core research groups. France, the United States, and Germany were consistently identified as the top contributors in both the overall virophage corpus and the marine sub-corpus (Table 2), indicating that the conceptual formation and expansion of virophage research have been closely linked to North American and European research infrastructures and collaboration networks. In particular, France played a pivotal role in establishing the field’s conceptual foundations through the discovery of *Mimivirus* and the Sputnik virophage [1,25]. Subsequently, other core countries, including the United States and Germany, contributed to expanding the themes toward giant viruses, mobile genetic elements, and the evolutionary relationships among satellite viruses [2,4].

Author co-authorship network analysis further revealed that a small number of central researchers exhibit high network centrality and mediate extensive collaboration ties, with clusters forming around these hubs (Figure 4). Such a structure may have been advantageous during the early stages of the field by promoting conceptual coherence, methodological standardization, and the consolidation of classification and definitional frameworks. However, it may also impose structural constraints on thematic diversification, host-range expansion, and the incorporation of broader environmental contexts. This limitation is particularly relevant to marine virophage research, which relies heavily on large-scale environmental sampling, high-performance computing, and advanced metagenomic analysis. When research leadership is concentrated in specific regions or institutions, observational targets and interpretive frameworks may become biased. The relatively more distributed connectivity observed in the country collaboration network of the marine subdomain, compared with the overall corpus (Figure 5), suggests that marine research depends more strongly on international data-sharing and multi-institutional collaboration infrastructures. Accordingly, future expansion and validation of virophage research will benefit from strategies that leverage existing hub-centered systems while actively promoting diversification of data production and research leadership through broader international collaboration.

### 4.5. Development of the Intellectual Structure: From Model Systems to Data-Centered Research

Keyword co-occurrence networks, frequency analyses, thematic maps, and thematic evolution analyses (Figure 6, Figure 7 and Figure 8, Figures S5 and S6; Table S4) consistently indicate that the intellectual structure of virophage research has shifted over time from a model system–centered framework toward a data-centered paradigm driven by environmental genomics and computational analysis. In the overall corpus, keywords such as virophage, giant virus, *Mimivirus*, and NCLDV formed the core of the network, reflecting the dominance of amoeba–giant dsDNA virus experimental models in early research (Figure 6A). In contrast, the marine subdomain exhibited relatively higher centrality and density for keywords such as metagenomics, genome sequence, alignment, polinton, and horizontal gene transfer (Figure 6B and 7B), highlighting the central role of environmental genomic discovery, sequence-based classification, and evolutionary inference [6,7].

The emergence of polinton-like viruses (PLVs) and related mobile genetic elements as niche or motor themes in the marine subdomain (Figure 7B) indicates an expansion of research questions beyond mechanistic analyses within single experimental models toward the interpretation of long-term evolutionary relationships linking eukaryotic viruses, mobile genetic elements, and host genomes [3]. Thematic evolution analysis further supports this distinction (Figure 8). While the overall corpus maintains continuity with early taxonomic and model-system–based themes across successive periods, the marine subdomain exhibits a more pronounced reorganization of thematic trajectories, centered on environmental context, genome-analytic tools, and evolutionary interpretation (Figure 8). This pattern reflects a shift from definition-oriented questions (“What is a virophage?”) toward system-level inquiries (“Where do virophages occur, how prevalent are they, in what forms do they exist, and what evolutionary roles might they play?”). Accordingly, marine virophage research can be interpreted not merely as an environmental application of the field, but as a catalytic domain that repositions virophages from accessory satellites of giant viruses to central nodes within broader evolutionary networks.

### 4.6. Ecological and Evolutionary Implications and Future Research Directions

These intellectual and thematic transitions position marine virophages not at the periphery of giant virus research, but as key components in reinterpreting marine viral ecosystems and the evolution of eukaryotic microorganisms. Virophages can indirectly influence host survival, population dynamics, and community structure by inhibiting or modulating giant virus replication [12,26], and the *Mavirus* system demonstrates that such functional switches can be experimentally validated rather than remaining purely hypothetical [2]. Nevertheless, a substantial proportion of marine virophage research remains predictive and metagenomics-driven, with limited experimental validation of infection cycles, host specificity, and ecological impacts. This trend is also reflected in the bibliometric results, which reveal a shift from early discovery-based studies to metagenomics-driven environmental virology. Future research would therefore benefit from strengthening two parallel and complementary tracks. First, a computational track focused on systematic discovery, refined annotation, functional prediction, and prioritization of candidate virophages from large-scale environmental genomic datasets. Second, an experimental track aimed at validating infection mechanisms, host ranges, and ecological effects for prioritized candidates through cultivation-based infection assays and controlled experiments [24]. In particular, the expansion of cultivation and infection systems for marine algae and protists is likely to be a critical prerequisite for transitioning marine virophage research from a prediction-oriented stage to a validation-oriented stage.

### 4.7. Limitations and Scope

This study has several limitations inherent to bibliometric analyses based on the Web of Science Core Collection. Because the quantitative corpus was constructed strictly using a predefined WoSCC search strategy, research published in regional journals not indexed in WoS, preprints, non-traditional publication formats, and very recent articles indexed in other databases but not yet retrievable in WoSCC may be underrepresented. Although the literature search was conducted on 6 January 2026, the analytical corpus included only publications from 2008 to 2025 that were retrievable in WoSCC at the time of data collection. This limitation is particularly relevant to rapidly emerging computational studies focused on the metagenomic recovery of giant viruses, polinton-like viruses, and virophages, and highlights the need for future updates using expanded databases and updated search strategies. In addition, keyword-based analyses are structurally sensitive to heterogeneity in terminology, shifts in classification systems, and biases in author-driven keyword selection. Citation-based interpretations should also be made cautiously, because cumulative citation counts may be influenced by publication age and because some highly cited studies in the dataset address broader aquatic virus research rather than virophages exclusively. Therefore, citation counts in this study should be interpreted as indicators of scholarly visibility within the broader virophage-related research context, rather than as direct measures of article quality or citations dedicated solely to virophage biology. Nevertheless, by applying identical data collection, preprocessing, and analytical criteria to both the overall virophage corpus and the marine subdomain, and by transparently reporting the analytical workflow [16], this study provides a reliable comparative framework for assessing the relative structures, growth patterns, and intellectual transitions of the two domains.

## 5. Conclusions

Using a bibliometric analysis of the Web of Science Core Collection, this study mapped the development of virophage research from 2008 to 6 January 2025, with a focused comparison between the overall virophage corpus and the marine virophage subdomain. The results indicate a shift from early discovery-based studies toward data-driven research shaped by environmental genomics and computational analysis. Although marine virophage research remains smaller in scale, it has shown notable citation impact and thematic distinctiveness. Overall, marine studies have contributed substantially to broadening the ecological and evolutionary understanding of virophages within viral and mobile genetic element networks.

## Figures and Tables

**Table 1:** Descriptive statistics of virophage research (2008–2025). Summary of core bibliometric indicators for the overall virophage research corpus (GVC) and the marine virophage sub-corpus (MVC).

Item	Overall Virophage Research	Marine Virophage Research
Final number of articles included (*n*)	221	49
Analysis period	2008–2025	2008–2025
Total citations (times)	10,434	1642
Average citations per article	47.21	33.5
Average number of articles per year (*n*/year)	12.3	2.7
Proportion of total (%)	100	22.2

**Table 2:** Country-level publication output in virophage research. Number of publications and proportional contributions (%) by country for overall virophage research (*n* = 221) and marine virophage research (*n* = 49) over the study period.

Rank	Country	Global Virophage Publications (n)	Share of Global Total (%)	Marine Virophage Publications (n)	Share of Marine Total (%)
1	France	103	46.2	11	22.4
2	USA	66	29.6	16	32.7
3	Germany	36	16.1	13	26.5
4	China	17	7.6	7	14.3
5	Brazil	17	7.6	1	2.0
6	UK	15	6.7	7	14.3
7	Canada	13	5.8	3	6.1
8	Netherlands	11	4.9	7	14.3
9	Japan	11	4.9	5	10.2
10	Poland	9	4.0	2	4.1
	Total (papers)	221		49	

## Data Availability

No datasets were generated or analyzed during the current study.

## Supplementary Materials

The following supporting information can be downloaded at: https://www.mdpi.com/article/10.3390/v18050560/s1, Supplementary data: The dataset of published literature; Figure S1: Annual publication trends and relative contribution of marine virophage research (2008–2025); Figure S2: Annual citation dynamics and impact trajectories in virophage research (2008–2025); Figure S3: Geographical distribution of research leadership: Corresponding and first authors in virophage research; Figure S4: Lexical landscape and thematic salience in virophage research; Figure S5: Temporal trends and thematic shifts in major keywords; Figure S6: Country–keyword coupling networks and national thematic specialization; Table S1: Detailed literature search strategy and criteria for corpus construction; Table S2: High-impact contributions: Top 20 most cited articles in the Global Virophage Corpus (GVC); Table S3: Domain-specific impact: Top 20 most cited articles in the Marine Virophage Sub-corpus (MVC); Table S4: Comparative analysis of keyword prominence: Top 20 author keywords in GVC and MVC.
