# Supplementary material for: Biological Trajectory of Virophage Research and the Emergence of Marine Virophages: A Scoping Review
Source: Viruses. 2026 May 14;18(5):560. doi: 10.3390/v18050560 (PMC13211728; doi:10.3390/v18050560)
Supplement: Supplementary file 1 [file viruses-18-00560-s001.zip › 20260513_Supplementary Information.pdf]

Supplementary Information

Supplementary Tables

Table S1. Detailed literature search strategy and criteria for corpus construction. Comprehensive summary of the search strings and systematic filtering process used to develop the Global Virophage Corpus (GVC) and the Marine Virophage Sub-corpus (MVC)

(A) Literature search queries

Category	Database	Search field	Search query
Overall virophage research	Web of Science Core Collection	Topic (TS)	TS = (virophage* OR "viro-phage" OR "virophage-like" OR "Sputnik virus" OR MavirusOR "transpovirion" OR "Polinton-like virus*" OR "Polintonvirus*")
Marine virophage research	Web of Science Core Collection	Topic (TS)	TS = ((virophage* OR "viro-phage" OR "virophage-like" OR "Sputnik virus" OR MavirusOR "transpovirion" OR "Polinton-like virus*" OR "Polintonvirus*") AND (marine OR ocean* OR seawater OR pelagic OR estuar* OR plankton OR coastal OR shelf OR virome* OR metagenom*))

(B) Exclusion criteria

Step	Exclusion criterion	Description
1	Exclude by document type	Proceedings Paper, Correction, Letter, Meeting Abstract, News Item
2	Exclude irrelevant studies	Articles in which virophage-related terms are not identified in the Abstract, Author Keywords, or Keywords Plus
3	Remove duplicates	Duplicate records with the same DOI or the same bibliographic information
4	Exclude non-marine studies (for the marine subset)	For the marine virophage subset, exclude articles that do not show a direct association with marine environments (e.g., ocean/coastal areas, seawater, plankton, or marine metagenomes)

(C) Number of articles included in the Final analysis

Category	Initial search results (n)	Final included articles after exclusions (n)
Overall virophage research	247	221
Marine virophage research	97	49

Table S2. High-impact contributions: Top 20 most cited articles in the Global Virophage Corpus (GVC). A ranked list of the 20 most influential publications in overall virophage research (2008–2025) based on cumulative citation counts.

Rank	Article Title	Journal	Publication Year	Total citations
1	VirSorter2: a multi-classifier, expert-guided approach to detect diverse DNA and RNA viruses	MICROBIOME	2021	780
2	Single mimivirus particles intercepted and imaged with an X-ray laser	NATURE	2011	735
3	Virus taxonomy in the age of metagenomics	NATURE REVIEWS MICROBIOLOGY	2017	542
4	The virophage as a unique parasite of the giant mimivirus	NATURE	2008	425
5	Giant Marseillevirus highlights the role of amoebae as a melting pot in emergence of chimeric microorganisms	PROCEEDINGS OF THE NATIONAL ACADEMY OF SCIENCES OF THE UNITED STATES OF AMERICA	2009	321
6	Distant Mimivirus relative with a larger genome highlights the fundamental features of Megaviridae	PROCEEDINGS OF THE NATIONAL ACADEMY OF SCIENCES OF THE UNITED STATES OF AMERICA	2011	257
7	Eukaryotic large nucleo-cytoplasmic DNA viruses: Clusters of orthologous genes and reconstruction of viral genome evolution	VIROLOGY JOURNAL	2009	250
8	Virophage control of antarctic algal host-virus dynamics	PROCEEDINGS OF THE NATIONAL ACADEMY OF SCIENCES OF THE UNITED STATES OF AMERICA	2011	231
9	A Virophage at the Origin of Large DNA Transposons	SCIENCE	2011	216
10	Provirophages and transpovirons as the diverse mobilome of giant viruses	PROCEEDINGS OF THE NATIONAL ACADEMY OF SCIENCES OF THE UNITED STATES OF AMERICA	2012	164
11	Genome of Phaeocystis globosa virus PgV-16T highlights the common ancestry of the largest known DNA viruses infecting eukaryotes	PROCEEDINGS OF THE NATIONAL ACADEMY OF SCIENCES OF THE UNITED STATES OF AMERICA	2013	156
12	Polintons: a hotbed of eukaryotic virus, transposon and plasmid evolution	NATURE REVIEWS MICROBIOLOGY	2015	147
13	Mimivirus and its Virophage	ANNUAL REVIEW OF GENETICS	2009	138
14	Reclassification of Giant Viruses Composing a Fourth Domain of Life in the New Order Megavirales	INTERVIROLOGY	2012	133
15	Evolution of the Large Nucleocytoplasmic DNA Viruses of Eukaryotes and Convergent Origins of Viral Gigantism	ADVANCES IN VIRUS RESEARCH	2019	126
16	Mimivirus shows dramatic genome reduction after intraamoebal culture	PROCEEDINGS OF THE NATIONAL ACADEMY OF SCIENCES OF THE UNITED STATES OF AMERICA	2011	116
17	mRNA deep sequencing reveals 75 new genes and a complex transcriptional landscape in Mimivirus	GENOME RESEARCH	2010	116
18	Tentative Characterization of New Environmental Giant Viruses by MALDI-TOF Mass Spectrometry	INTERVIROLOGY	2010	110

19	Mimivirus: leading the way in the discovery of giant viruses of amoebae	NATURE REVIEWS MICROBIOLOGY	2017	108
20	Host genome integration and giant virus-induced reactivation of the virophage mavirus	NATURE	2016	108

Table S3. Domain-specific impact: Top 20 most cited articles in the Marine Virophage Sub-corpus (MVC).

A ranked list of the 20 most cited publications within the marine virophage subdomain (2008–2025)

Rank	Article Title	Journal	Publication Year	Total citations
1	The virophage as a unique parasite of the giant mimivirus	NATURE	2008	425
2	Virophage control of antarctic algal host-virus dynamics	PROCEEDINGS OF THE NATIONAL ACADEMY OF SCIENCES OF THE UNITED STATES OF AMERICA	2011	231
3	Genome of Phaeocystis globosa virus PgV-16T highlights the common ancestry of the largest known DNA viruses infecting eukaryotes	PROCEEDINGS OF THE NATIONAL ACADEMY OF SCIENCES OF THE UNITED STATES OF AMERICA	2013	156
4	Virus Genomes from Deep Sea Sediments Expand the Ocean Megavirome and Support Independent Origins of Viral Gigantism	MBIO	2019	94
5	Analysis of virus genomes from glacial environments reveals novel virus groups with unusual host interactions	FRONTIERS IN MICROBIOLOGY	2015	69
6	Diversity, evolutionary contribution and ecological roles of aquatic viruses	SCIENCE CHINA-LIFE SCIENCES	2018	62
7	High-throughput isolation of giant viruses of the Mimiviridae and Marseilleviridae families in the Tunisian environment	ENVIRONMENTAL MICROBIOLOGY	2013	58
8	Mimiviridae: An Expanding Family of Highly Diverse Large dsDNA Viruses Infecting a Wide Phylogenetic Range of Aquatic Eukaryotes	VIRUSES-BASEL	2018	57
9	Virophages and retrotransposons colonize the genomes of a heterotrophic flagellate	ELIFE	2021	41
10	Polinton-like viruses are abundant in aquatic ecosystems	MICROBIOME	2021	40
11	Isolation and infection cycle of a polinton-like virus virophage in an abundant marine alga	NATURE MICROBIOLOGY	2023	33
12	The Giant Virus Finder discovers an abundance of giant viruses in the Antarctic dry valleys	ARCHIVES OF VIROLOGY	2017	26
13	Impact of external forces on cyanophage-host interactions in aquatic ecosystems	WORLD JOURNAL OF MICROBIOLOGY & BIOTECHNOLOGY	2013	26
14	Quantitative Assessment of Nucleocytoplasmic Large DNA Virus and Host Interactions Predicted by Co-occurrence Analyses	MSPHERE	2021	25
15	Diverse and unique viruses discovered in the surface water of the East China Sea	BMC GENOMICS	2020	25
16	Metagenomic Characterization of the Viral Community of the South Scotia Ridge	VIRUSES-BASEL	2019	25
17	Isolation and Identification of a Large Green Alga Virus (Chlorella Virus XW01) of Mimiviridae and Its Virophage (Chlorella Virus Virophage SW01) by Using Unicellular	JOURNAL OF VIROLOGY	2022	21

	Green Algal Cultures			
18	Four high-quality draft genome assemblies of the marine heterotrophic nanoflagellate Cafeteria roenbergensis	SCIENTIFIC DATA	2020	19
19	Development of DNA mismatch repair gene, MutS, as a diagnostic marker for detection and phylogenetic analysis of algal Megaviruses	VIROLOGY	2014	16
20	Capsid protein structure, self-assembly, and processing reveal morphogenesis of the marine virophage mavirus	PROCEEDINGS OF THE NATIONAL ACADEMY OF SCIENCES OF THE UNITED STATES OF AMERICA	2018	14

Table S4. Comparative analysis of keyword prominence: Top 20 author keywords in GVC and MVC. Frequency (n) and relative proportion (%) of the 20 most frequent author keywords within the Global Virophage Corpus (GVC) and the Marine Virophage Sub-corpus (MVC)

Rank	Keyword	Global virophage publications (n)	Share of global total (%)	Marine virophage publications (n)	Share of marine total (%)
1	Virophage	51	22.9	8	16.3
2	Giant Virus	32	14.3	4	8.2
3	Mimivirus	32	14.3	0	0.0
4	Giant Viruses	19	8.5	2	4.1
5	Virophages	18	8.1	3	6.1
6	Megavirales	13	5.8	0	0.0
7	Marseillevirus	11	4.9	0	0.0
8	Metagenomics	10	4.5	4	8.2
9	NCLDV	10	4.5	3	6.1
10	Mimiviridae	10	4.5	2	4.1
11	Virus	8	3.6	3	6.1
12	Viruses	8	3.6	3	6.1
13	Amoeba	8	3.6	0	0.0
14	Evolution	7	3.1	1	2.0
15	Polinton	6	2.7	3	6.1
16	Nucleocytoplasmic Large DNA Viruses	6	2.7	1	2.0
17	Diversity	5	2.2	4	8.2
18	Horizontal Gene Transfer	5	2.2	3	6.1
19	Virus Evolution	5	2.2	2	4.1
20	Polintons	5	2.2	1	2.0

Supplementary Figures

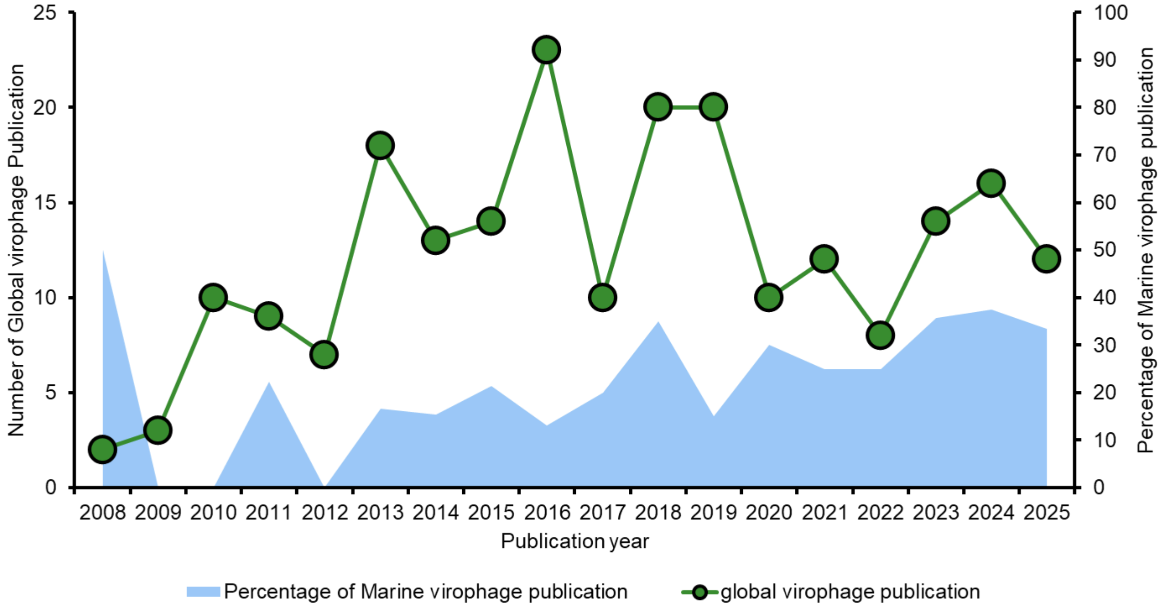

Fig. S1. Annual publication trends and relative contribution of marine virophage research (2008–2025). Dual-axis visualization showing the absolute annual publication output of the Global Virophage Corpus (GVC; green line) and the corresponding proportional contribution (%) of the Marine Virophage Sub-corpus (MVC; blue area).

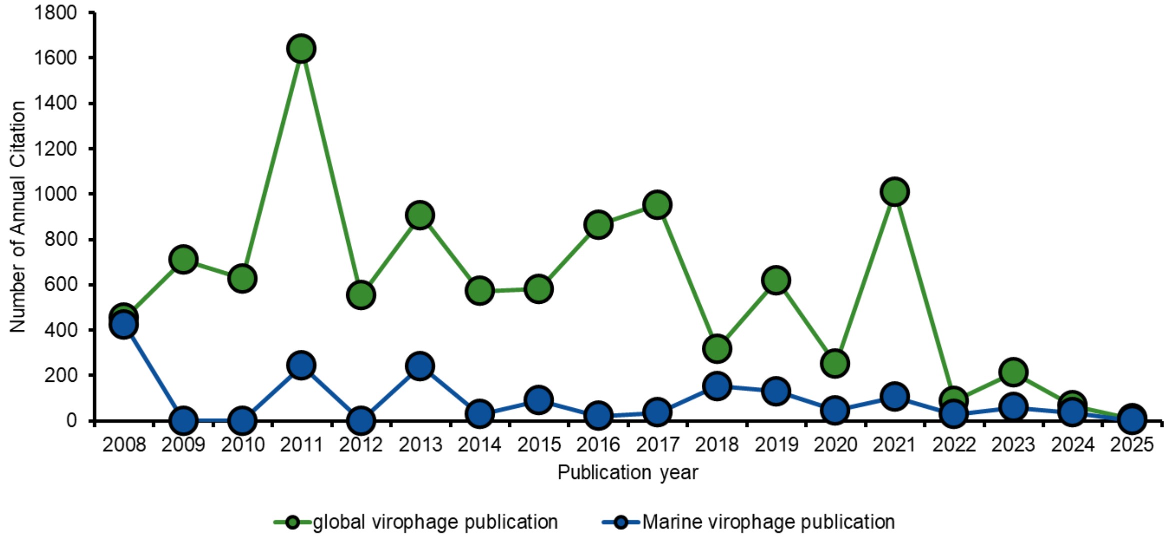

Fig. S2. Annual citation dynamics and impact trajectories in virophage research (2008–2025). Comparative visualization of annual total citation counts for the Global Virophage Corpus (GVC; green line) and the Marine Virophage Sub-corpus (MVC; blue line).

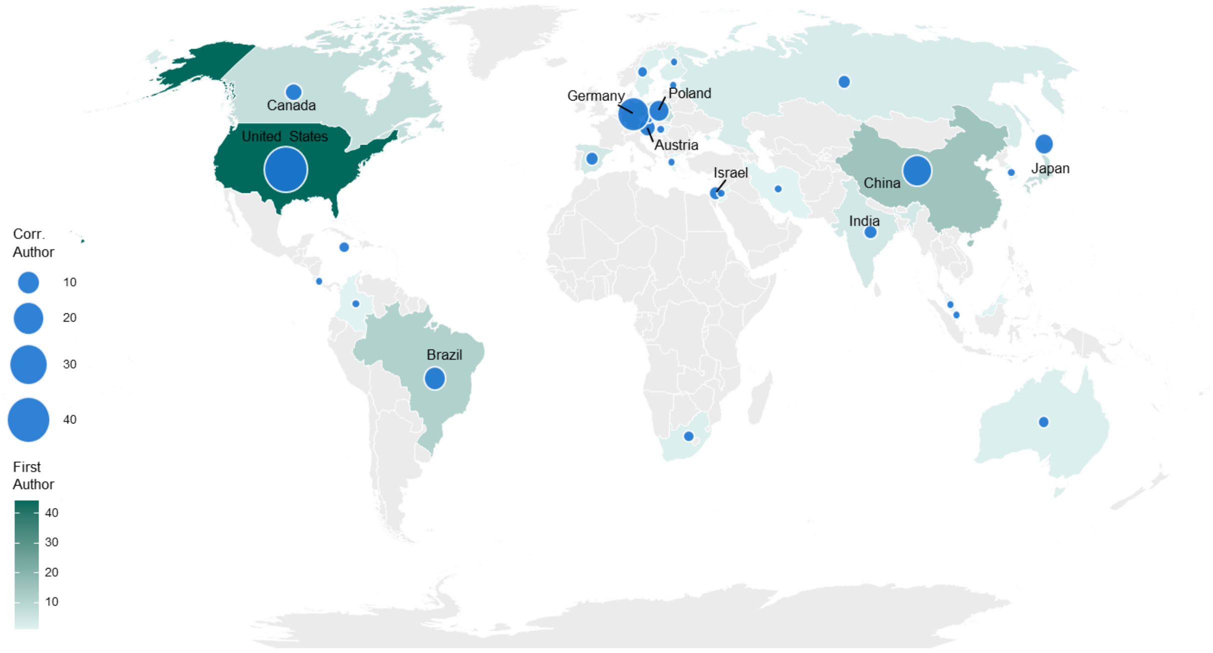

Fig. S3. Geographical distribution of research leadership: Corresponding and first authors in virophage research. Global map illustrating the national distribution of research leadership based on author roles.

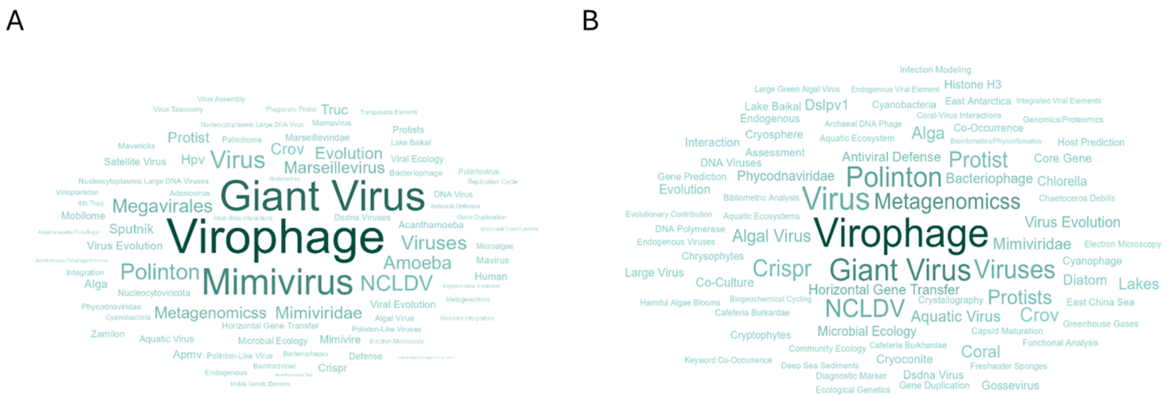

Fig. S4. Lexical landscape and thematic salience in virophage research. Author keyword word clouds for (A) the Global Virophage Corpus (GVC) and (B) the Marine Virophage Sub-corpus (MVC).

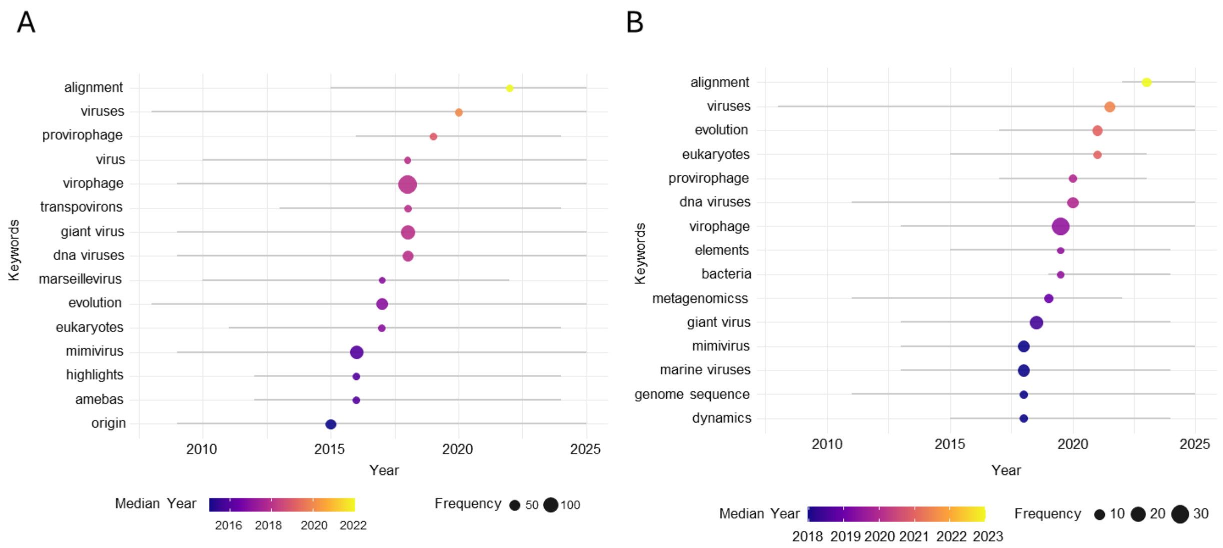

Fig. S5. Temporal trends and thematic shifts of major keywords. Visualization of the temporal distribution of top author keywords for (A) the Global Virophage Corpus (GVC) and (B) the Marine Virophage Sub-corpus (MVC).

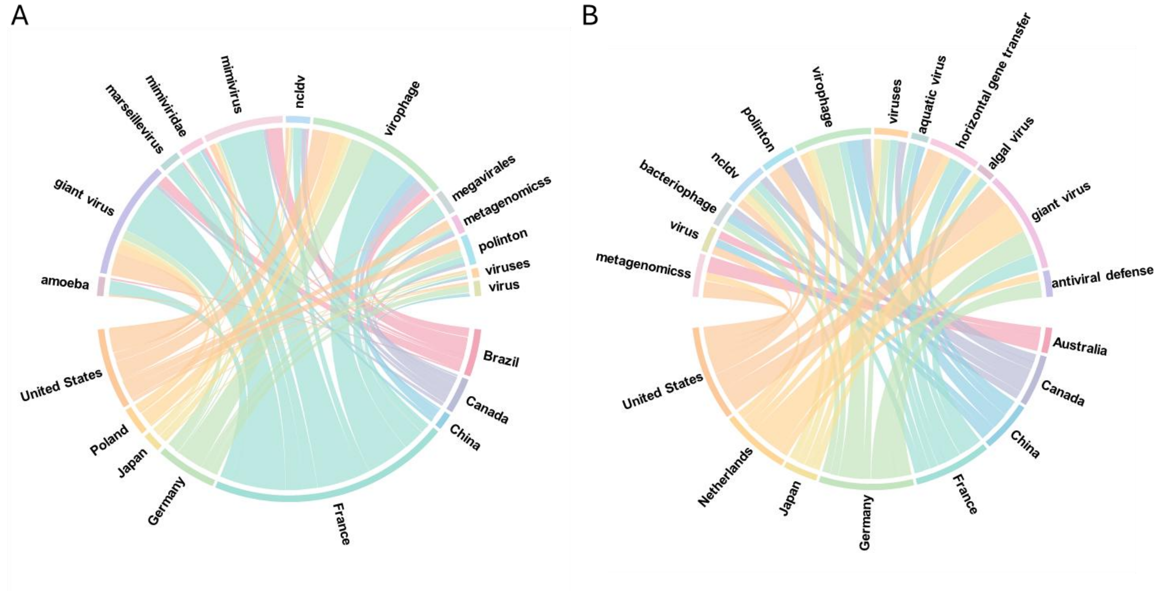

Fig. S6. Country–keyword coupling networks and national thematic specialization. Chord diagrams illustrating the associative relationships between major contributing countries and top author keywords for (A) the Global Virophage Corpus (GVC) and (B) the Marine Virophage Sub-corpus (MVC).
